# A Design of Small Area, 0.95 mW, 612–1152 MHz Open Loop Injection-Locked Frequency Multiplier for IoT Sensor Applications

**DOI:** 10.3390/s18061777

**Published:** 2018-06-01

**Authors:** SungJin Kim, Dong-Gyu Kim, Chanho Kim, Dong Soo Lee, Behnam Samadpoor Rikan, YoungGun Pu, Sang-Sun Yoo, Minjae Lee, KeumCheol Hwang, Youngoo Yang, Kang-Yoon Lee

**Affiliations:** 1 College of Information and Communication Engineering, Sungkyunkwan University, Suwon 16419, Korea; sun107ksj@skku.edu (S.J.K.); rlarlarbrb@skku.edu (D.-G.K.); muser49@skku.edu (C.K.); blacklds@skku.edu (D.S.L.); behnam@skku.edu (B.S.R.); hara1015@naver.com (Y.G.P.); khwang@skku.edu (K.C.H.); yang09@skku.edu (Y.Y.); 2 Department of Smart Automobile, Pyeongtaek University, Pyeongtaek 17869, Korea; rapter@kaist.ac.kr; 3 School of Information and Communications, Gwangju Institute of Science and Technology, Gwangju 61005, Korea; minjae@gist.ac.kr

**Keywords:** injection locked frequency multiplier, frequency locked loop (FLL), phase noise

## Abstract

This paper presents a 612–1152 MHz Injection-Locked Frequency Multiplier (ILFM). The proposed ILFM is used to send an input signal to a receiver in only the I/Q mismatch calibration mode. Adopting a Phase-Locked Loop (PLL) to calibrate the receiver places a burden on this system because of the additional area and power consumption that is required. Instead of the PLL, to satisfy high-frequency, low-jitter and low-area requirements, a Ring Oscillator is adopted in the system. The free-running frequency of the ILFM is automatically and digitally calibrated to reflect the frequency of the injected signal from the harmonics of the reference clock. To control the frequency of the ILFM, the load current is digitally tuned with a 6-bit digital control signal. The proposed ILFM locks to the target frequency using a digitally controlled Frequency Locked Loop (FLL). This chip is fabricated using 1-poly 6-metal 0.18 µm CMOS and has achieved the wide tuning range of 612–1152 MHz. The power consumption is 0.95 mW from a supply voltage of 1.8 V. The measured phase noise of the ILFM is −108 dBc/Hz at a 1 MHz offset.

## 1. Introduction

**Figure 1:**
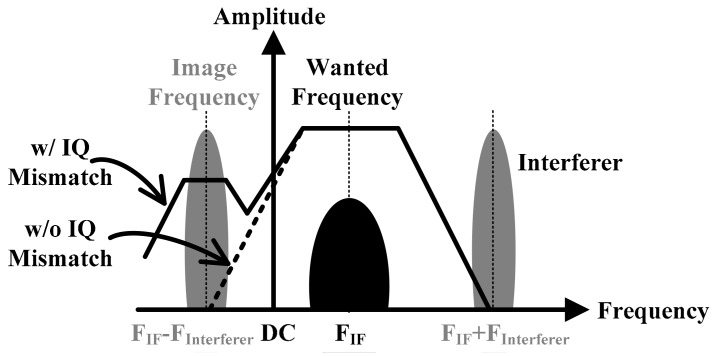
AC characteristics of low-IF receiver when I/Q mismatch occurs.

Recently, the Internet of Things (IoT) has been applied to many applications, such as sensor networks and wearable devices. In these applications, low power consumption and small chip area are required to increase battery life and reduce system cost. Therefore, Integrated Chip (IC)s for IoT sensors should be designed to meet these requirements. Low-Intermediate Frequency (IF) receiver architectures have become popular for low-power applications. These structures offer advantages over Zero-IF architectures in terms of the DC-Offset calibration and flicker noise [1]. In low-IF architectures, the down-converted complex baseband signal is represented by two real In-phase and Quadrature-phase (I/Q) signals. Analog parameter variations in the local oscillators, mixer and filters result in gain and phase errors between I/Q paths. Due to these errors, image leaks into the signal band during the down-conversion process. Therefore, the low-IF receiver has a low image rejection ratio (IRR), as shown in Figure 1 [2].

**Figure 2:**
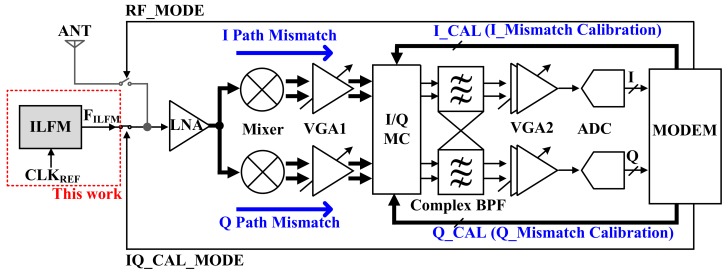
The block diagram of Low IF receiver with an Injection-Locked Frequency Multiplier (ILFM).

Methods for solving image problems by compensating I/Q mismatch are presented in References [2,3,4,5,6]. During the I/Q mismatch calibration phase, the Injection-Locked Frequency Multiplier (ILFM) block generates the same frequency as the RF signal before receiving the RF signal through the antenna, as shown Figure 1. Since the I/Q signals in the low-IF receiver structure flow to the I/Q signal in the band pass filter (BPF), the I/Q mismatch of the BPF input becomes dominant. Therefore, it is effective to add I/QMC (I/Q Mismatch Calibrator) to compensate for the mismatch of I/Q signals before the BPF. Figure 2 shows a block diagram of the whole low IF using ILFM and I/QMC.

A subharmonic ILFM has been considered as a promising solution to generate low phase noise and high-frequency clocks using a limited silicon area and power consumption budget. However, previous ILFMs are highly sensitive to process, voltage and temperature (PVT) variations. Recently, several papers have been published that suggest some approaches to overcome this problem [7,8,9,10,11,12,13,14,15,16,17,18,19,20,21,22,23,24,25]. In References [10,11,12,13], the structure using the most basic single-loop PLL or DLL is used. However, this structure cannot prevent the real-time frequency drift that occurs through supply voltage and temperature variations. In References [14,15,16,17,18,19], a dual-loop structure with a main oscillator and a replica oscillator structure is used to improve the disadvantages of single-loop structure. However, the previous ILFM had the disadvantage of having a large chip area and large power consumption due to its complex structure. In Section 2, the pros and cons of prior works are described and analyzed in detail. In this paper, we propose an ILFM with small chip area and low power consumption using Frequency Locked Loop (FLL).

## 2. Prior ILFM Structures

**Figure 3:**
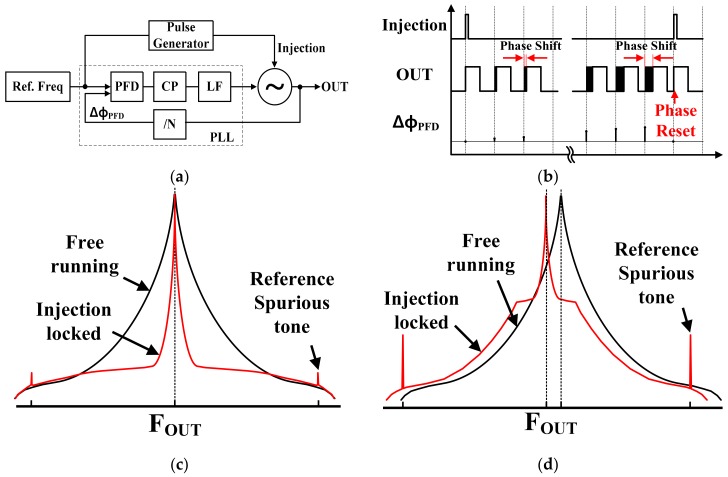
(**a**) Block diagram; (**b**) timing diagram of conventional ILFM; (**c**) phase noise of ILFM without frequency error; and (**d**) with frequency error.

Figure 3a is the block diagram of an ILFM structure with a PLL, and Figure 3b shows the timing diagram. As shown in Figure 3a,b, ILFM injects the reference clock into a Voltage Controlled Oscillator (VCO) and the injection signal realigns the output phase of the free-running VCO. Therefore, low phase noise performance is acquired [7]. This phase-realignment mechanism with the reference clock allows the ILFM to have low-jitter performance. Although the ILFM have many advantages, there are critical requirements for good phase noise performance. It can be achieved only when the target frequency of the ILFM is very close to the free-running frequency of the VCO. Therefore, a good phase noise performance of the ILFM might not be guaranteed, especially for a Ring Oscillator of which the free-running frequency is highly sensitive to PVT variation. In addition, if the frequency of the Ring Oscillator is out of the lock range of ILFM due to the PVT variation, ILFM cannot achieve the injection locking [8]. Therefore, ILFM typically requires an effective PVT calibrator or calibration methods to mitigate the sensitivity of performance to the PVT variation. Figure 3c shows the phase noise graph of the ILFM when the VCO frequency is close to the ILFM target frequency. On the other hand, Figure 3d is the phase noise graph of ILFM when the frequency of VCO changes due to the PVT variation. If there is a frequency error, the in-band phase noise performance is degraded, and the magnitude of reference spurious tone is increased [9].

**Figure 4:**
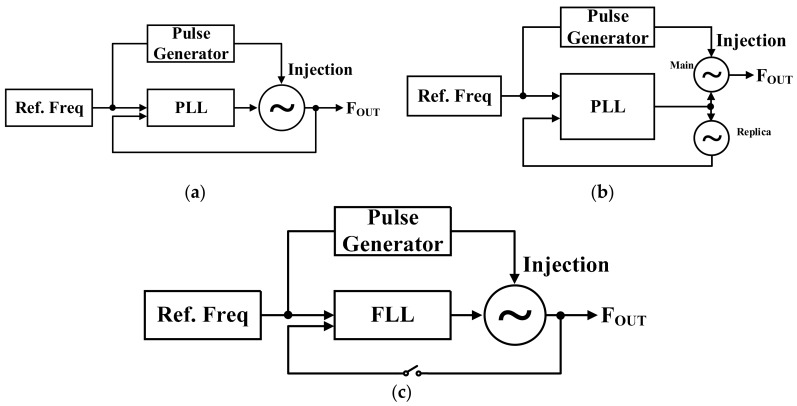
Structure of the ILFM (**a**) PLL based; (**b**) dual-loop PLL based; and (**c**) the proposed open-loop ILFM structure using FLL.

Conventional methods for overcoming PVT variations are shown in Figure 4a,b [9]. The most popular PVT calibration method is to use a PLL. Figure 4a shows a conventional ILFM structure with single-loop PLL [10,11,12]. The PLL is used to calibrate static PVT variation of the frequency of VCO. However, the structure cannot prevent the real-time frequency drift that occurs through supply voltage and temperature variations. This is because the structure has a PLL loop path and an injection path. A timing problem arises because the two paths operate independently [13]. To overcome this timing issue, an ILFM with two separate loops has been proposed. Figure 4b shows a dual-loop structure with the main oscillator and a replica oscillator [14,15,16,17,18,19,20]. The structure is proposed to resolve the timing problem of PLL based ILFM. The structure has two VCOs which are a main VCO and a replica VCO. The replica VCO is not injection-locked to prevent instantaneous frequency drift. The advantage of this method is that it can be calibrated with a frequency offset and PVT variation of the main VCO in real time using the replica VCO. However, if the mismatch occurs between main VCO and replica VCO, the structure cannot calibrate PVT variation. In addition, the implementation is difficult because it consists of two loops, and there is a disadvantage in that the die area and power consumption are doubled compared to a single structure [13]. Figure 4c shows the ILFM structure of an open loop type with FLL. Figure 4c can reduce the power consumption by turning off the FLL block after *F_OUT_* reaching the target frequency using FLL, and the chip area is small because only a simple FLL circuit is used.

## 3. Proposed Injection-Locked Frequency Multiplier (ILFM) with Frequency Tracking

**Figure 5:**
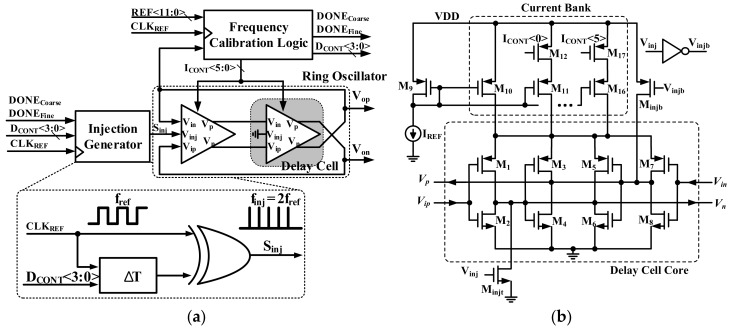
(**a**) Block diagram of proposed ILFM and (**b**) schematic of Delay Cell with injection switch.

Figure 5a shows a block diagram of the proposed ILFM. The proposed ILFM is composed of a Ring Oscillator, an Injection Generator, and FLL. Generally, the clock signal is required for calibration or other purposes in the transceiver or digital block. If the PLL is used to generate a clock signal, it will cause a burden on this system due to the additional area and power consumption. A Ring Oscillator is adopted instead of PLL to satisfy the high frequency and low area.

The Ring Oscillator generates a frequency which is close to the target frequency (f_TARGET_). If the output frequency of the ILFM (f_ILFM_) is near to the f_TARGET_, it is precisely locked to f_TARGET_ by the harmonics of the reference clock frequency (f_ref_), 36 MHz [7].

(1) $$f_{\mathrm{ILFM}}=M\times f_{\mathrm{ref}}$$

where f_ILFM_ is the output frequency of ILFM, and the multiplication factor *M* can be changed through the current control of the Delay Cell.

Figure 5b shows a schematic of the Delay Cell with an injection switch. The Delay Cell is composed of four inverters, a 6-bit Current Bank, and injection switches. A Ring Oscillator is proposed to acquire f_TARGET_, as shown in Figure 5a. The Current Bank is designed to adjust the frequency using Frequency Calibration Logic. Thus, it is possible to reduce PVT variations and improve phase noise. The injection switches of M_injt_, and M_injb_ are attached to the nodes of V_on_ and V_op_, respectively.

**Figure 6:**
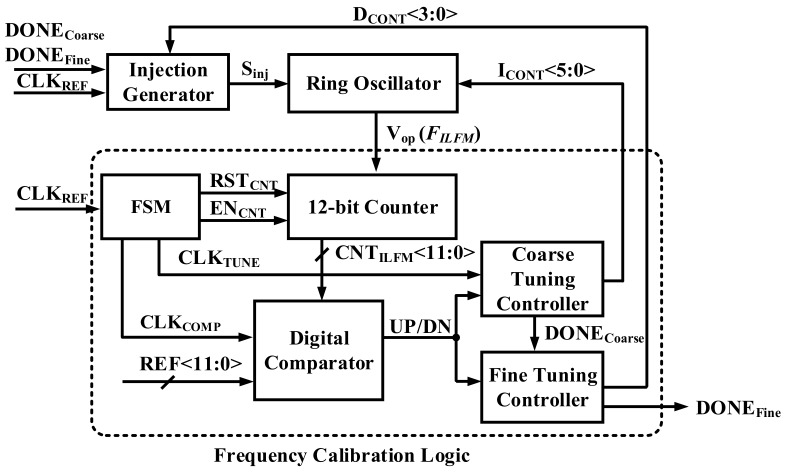
Block diagram of the Frequency Calibration Logic.

Figure 6 shows the Frequency Calibration Logic of ILFM. It is composed of a 12-bit Counter, a finite-state machine (FSM), a Digital Comparator, a Coarse Tuning Controller, and a Fine Tuning Controller. Since the ILFM has a locked f_TARGET_ from the Injection Generator, the free-running frequency must be close to the f_TARGET_. Therefore, the role of the Coarse Tuning Controller is to set the frequency close to f_TARGET_, by calibrating the free-running frequency of the Ring Oscillator. The role of the Fine Tuning Controller is to lock the ILFM to the f_TARGET_ by controlling the delay (∆T) of the injection generator. When the frequency calibration logic is started, the 12-bit counter counts the current frequency of the Ring Oscillator. The counted value CNT_ILFM_<11:0> is delivered to the digital comparator. Then, CNT_ILFM_<11:0> is compared to the reference number, REF<11:0>, which is determined from Equations (2) and (3) based on the f_TARGET_.

(2) $${T\_\mathrm{EN}}_{\mathrm{CNT}}(s)=\frac{1}{f_{\mathrm{ref}}(\mathrm{Hz})}\times 96$$

(3) $${\mathrm{REF}\;<0>T\_\mathrm{EN}}_{\mathrm{CNT}}(s)\times f_{\mathrm{TARGET}}(\mathrm{Hz})$$

where T_EN_CNT_ is the value of the interval in which the EN_CNT_ signal is High. f_ref_ is the reference clock frequency (36 MHz), and f_TARGET_ is the target frequency.

**Figure 7:**
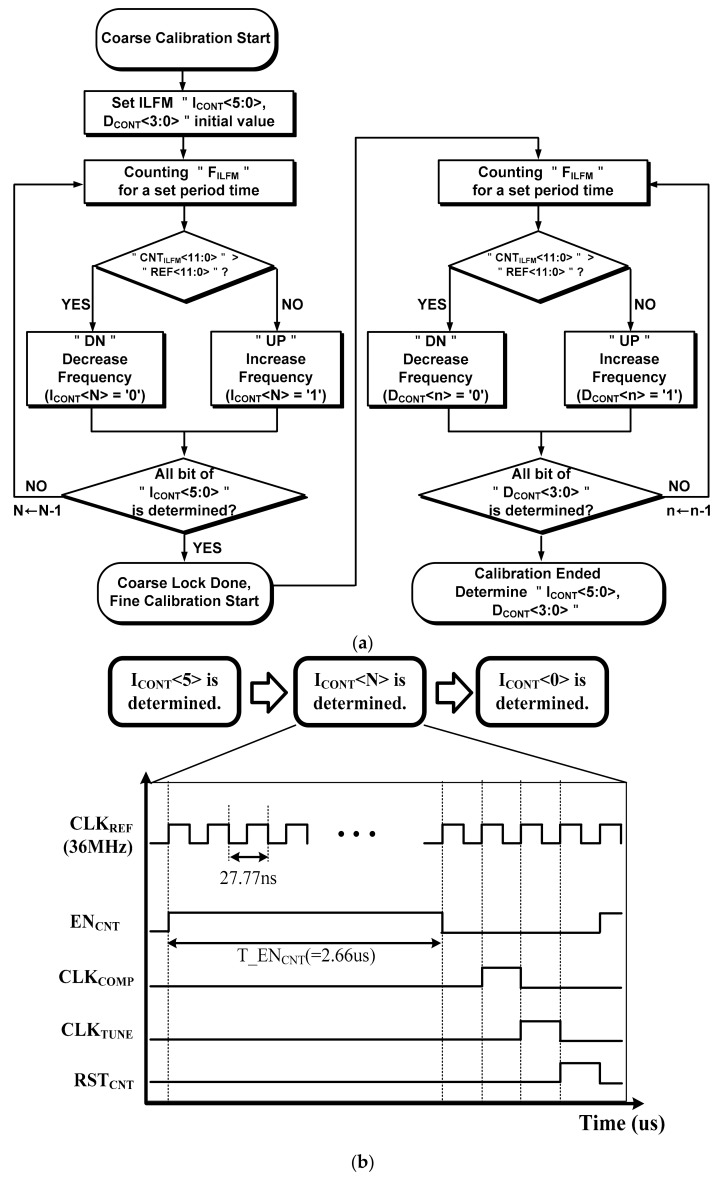
(**a**) Flow chart of Frequency Calibration Logic and (**b**) timing diagram of 1-state of FSM in Frequency Calibration Logic.

Figure 7a shows a flow chart of the Frequency Calibration Logic, and Figure 7b is a timing diagram of the FSM in the Frequency Calibration Logic. A 12-bit Counter is used to calculate the output frequency of the ILFM. It operates in an asynchronous way when a counter-enabled signal (EN_CNT_) is high and is periodically reset by the counter reset signal (RST_CNT_) generated by the FSM. The FSM determines the timing of the calibration by generating the decision clock (CLK_TUNE_) and a comparison clock (CLK_COMP_) using the CLK_REF_ signal [21].

If the value of CNT_ILFM_<11:0> is higher than REF<11:0>, the DN is generated by the Coarse Tuning Controller and Fine Tuning Controller. On the other hand, if the value of CNT_ILFM_<11:0> is lower than REF<11:0>, UP is generated. The calibration time is minimized by applying the binary search algorithm. Therefore, I_CONT_<5:0> is determined by the Coarse Tuning Controller after this loop has been operated six times. The fine tuning works in the same way as the coarse tuning, and the D_CONT_<3:0> values are determined when fine tuning is in progress. The D_CONT_<3:0> output determines the injection pulse width of the Injection Generator. The output frequency of the Ring Oscillator is sensitive to a PVT corner variation [7].

**Figure 8:**
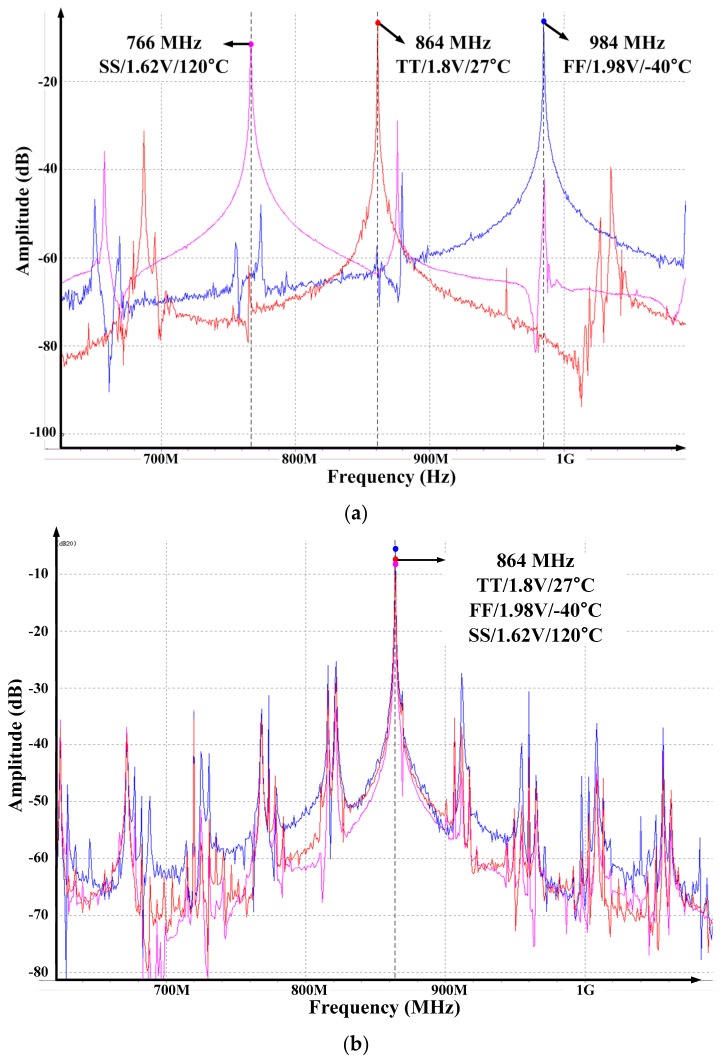
The simulation result of ILFM Frequency calibration (**a**) before calibration and (**b**) after calibration.

Figure 8a shows the simulation result of the frequency variation of the ILFM that is changed by the PVT variation. When frequency calibration logic is not used, the output frequency of the ILFM changes from 766 MHz to 948 MHz depending on the corner condition.

On the other hand, Figure 8b shows the simulation result when the proposed Frequency Calibration Logic is used, and the output frequency of ILFM is exactly calibrated to target frequency (846 MHz) at all PVT corner conditions.

## 4. Experimental Results

**Figure 9:**
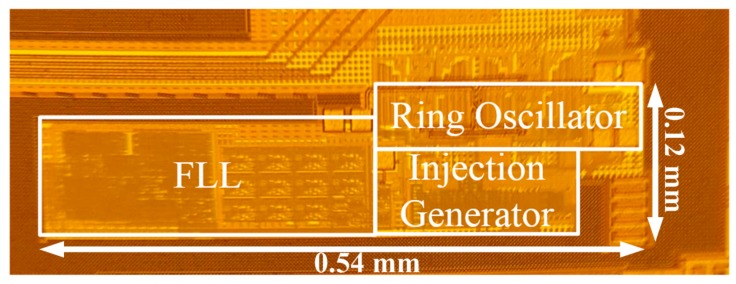
Chip microphotograph of ILFM.

Figure 9 shows a chip microphotograph of the ILFM. The proposed design is fabricated in a 0.18 μm CMOS process and the layout size of the entire ILFM including Ring Oscillator, Injection Generator and FLL is 0.54 mm × 0.12 mm.

**Figure 10:**
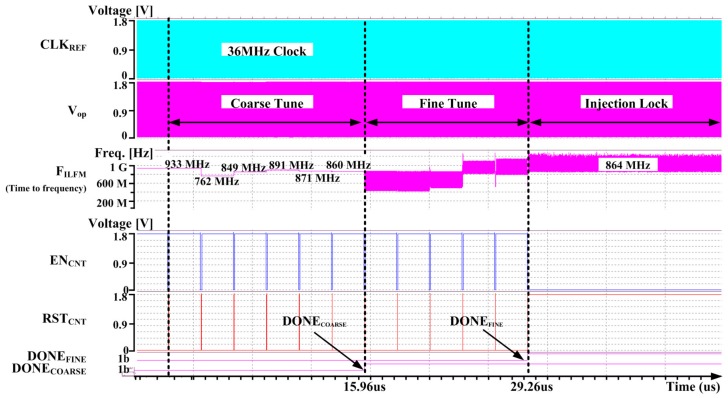
The top simulation of ILFM.

Figure 10 shows the transient simulation results when the target frequency of the ILFM is set as 864 MHz. In Figure 10, the output (V_op_) of Ring Oscillator and its frequency are plotted. The FLL tracks the target frequency using a successive approximation register (SAR) logic method. As it is shown in the flow chart in Figure 7a, during the FLL operation period, the frequency achieves the target frequency. After both of the DONE_Coarse_ and DONE_Fine_ signals change to ‘H’, the CLK_REF_ signal is injected and injection locked to the target frequency.

**Figure 11:**
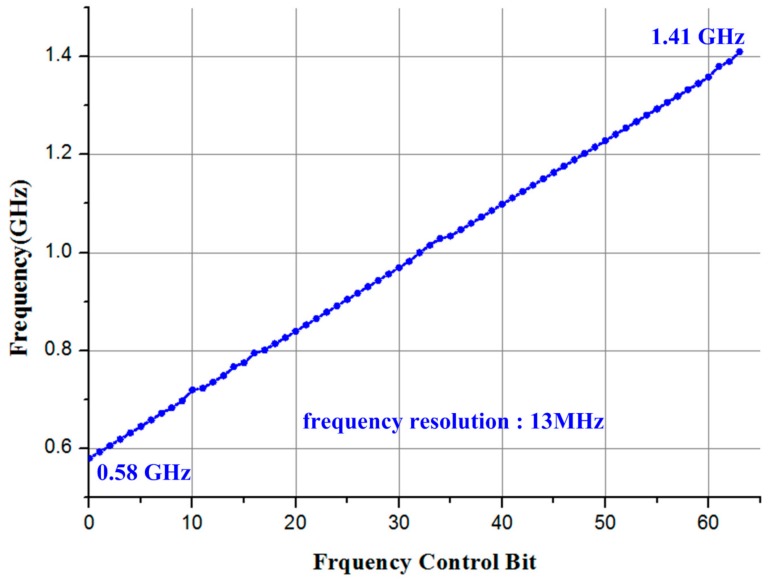
Free-running frequency range of Ring Oscillator.

Figure 11 shows the measurement results of the free-running frequency of the Ring Oscillator. The frequency of the ILFM is measured by changing I_CONT_<5:0> in the ILFM external control mode using a Keysight spectrum analyzer E4440A (Keysight, Santa Rosa, CA, USA). The frequency range of the Ring Oscillator can be adjusted with the Least Significant Bit (LSB) units of about 13 MHz and has a frequency range from 0.58 GHz to 1.41 GHz.

**Figure 12:**
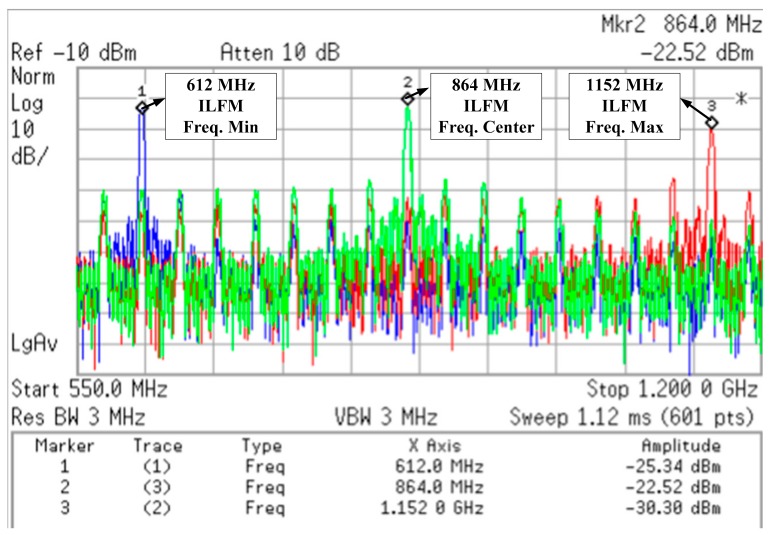
Injection Locked frequency range of ILFM.

Figure 12 shows the measured injection-locked full range of ILFM. It is measured using a Keysight spectrum analyzer E4440A. The output frequency of the ILFM ranges from 612 MHz to 1152 MHz, corresponding to 17 and 32 times 36 MHz, respectively. The ILFM can lock to N times the reference clock within the injection-locked range.

**Figure 13:**
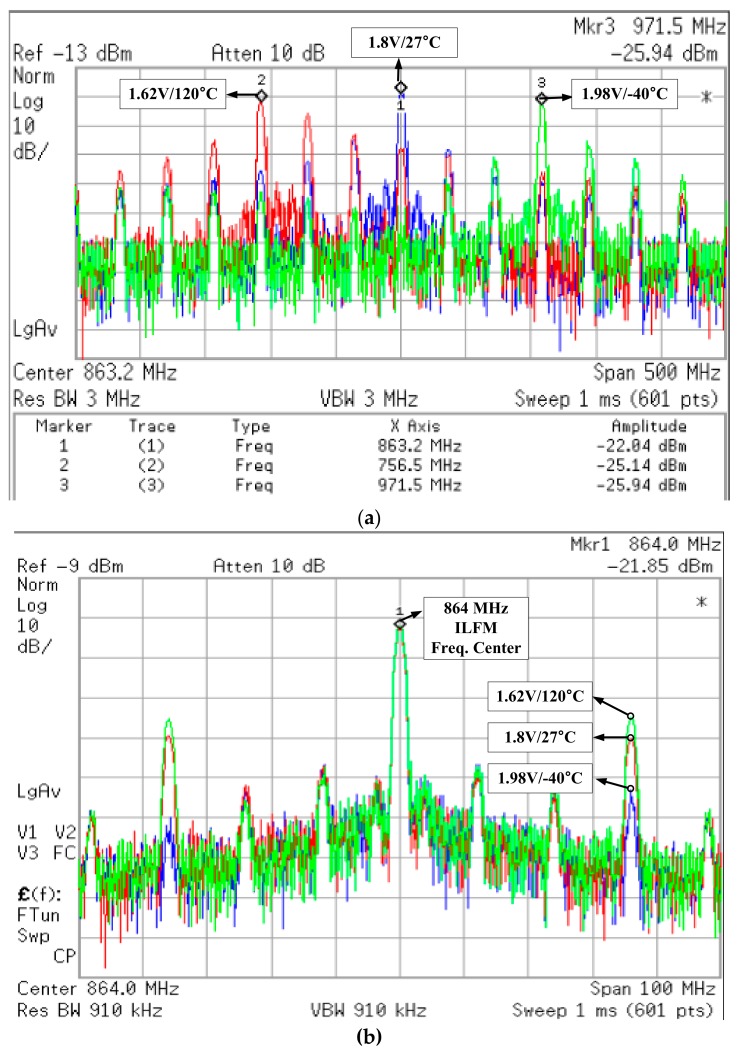
The measurement result of ILFM Frequency calibration (**a**) before calibration and (**b**) after calibration.

Figure 13a,b show the measured frequency variation of the ILFM that is changed by the supply voltage and temperature variation. It is measured using a Keysight spectrum analyzer E4440A. Figure 13a shows the result of spectrum measurement of ILFM without frequency calibration. When the voltage and the temperature change, the free-running frequency of the Ring Oscillator changes by about ±100MHz. Therefore, the output of the ILFM generates a large reference spurious tone, and the phase noise performance is not good. On the other hand, as Figure 13b shows, when the proposed Frequency Calibration Logic is used, the output frequency of ILFM is exactly calibrated to target frequency (846 MHz) at the supply voltage and temperature corner conditions.

**Figure 14:**
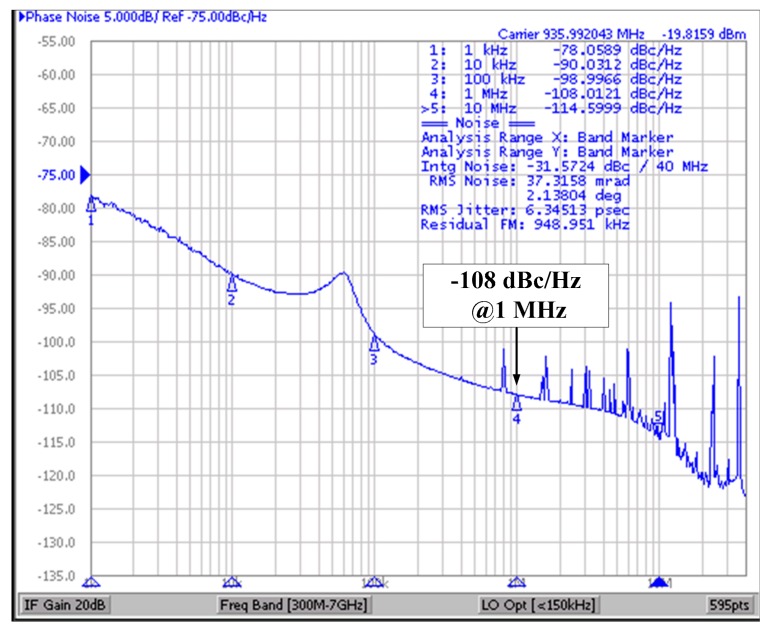
Measurement of injection-locked phase noise at center frequency.

Figure 14 shows the measured phase noise of the proposed ILFM using a Keysight signal source analyzer E5052A. When the short pulse is injected into the Ring Oscillator, the phase noise is −108 dBc/Hz at 1 MHz offset and the measured RMS jitter of the ILFM is 6.34 ps. When the ILFM is locked by injection, the phase noise is reduced more than the free-running noise. Furthermore, with the effect of the injection, in-band phase noise is also reduced.

Table 1 shows the comparison with published papers [22,23,24]. The proposed paper is designed to generate a RF signal to the RF frontend for calibration purpose before the RF signal input from the antenna. The RF signals for calibrating I/Q mismatch do not require ultra-low jitter performance. The reference spur is generated by the ILFM, and it is attenuated by the baseband filter. The performance of the proposed paper with the highest priority is the reduction of the silicon area and current consumption. The definition of *FOM* is defined as follows:

(4) $$FoM=10\cdot \log\left({\left(\frac{{Jitter}_{rms}}{1\;s}\right)}^{2}\cdot \left(\frac{P_{diss}}{1\;\mathrm{mW}}\right)\right)$$

where *Jitter_rms_*, 1 s, and *P_diss_* are the RMS jitter value of ILFM output, 1 s, and the power consumption, respectively [23].

## 5. Conclusions

This paper presents a 612–1152 MHz Injection-Locked Frequency Multiplier (ILFM). The proposed ILFM is used to send an input signal to the receiver only in the I/Q mismatch calibration mode. Adopting a PLL to calibrate the receiver places a burden on this system because additional area and power consumption are required. Instead of the PLL, to satisfy high-frequency, low-jitter and low-area requirements, a Ring Oscillator is adopted in the system. The free-running frequency of the ILFM is automatically and digitally calibrated to reflect the frequency of the injected signal from the harmonics of the reference clock. To control the frequency of the ILFM, the load current is digitally tuned with a 6-bit digital control signal. The proposed ILFM locks to the target frequency using a digitally controlled FLL. The proposed ILFM is designed for I/Q mismatch calibration in sub-GHz RF receivers. The advantage of this chip is the minimized area and current consumption that result from adopting a simple FLL logic. The FOM of this ILFM is not as good as the reference paper due to its low power consumption. However, in this application, area and power consumption are more important than RMS jitter performance. The reference spurious is filtered by the baseband filter stage. This chip is fabricated using 1-poly 6-metal 0.18 µm CMOS and has achieved the wide tuning range of 612~1152 MHz. The power consumption is 0.95 mW from a supply voltage of 1.8 V. The measured phase noise of the ILFM is −108 dBc/Hz at a 1 MHz offset.

## Figures and Tables

**Table 1:** Performance Comparison of Injection-Locked Frequency Multiplier (ILFM).

	[22]	[23]	[24]	This Work
Process (nm)	180 nm	65 nm	65 nm	180 nm
Topology	IL + Open Loop	IL + DPLL	IL + DPLL	IL + FLL
Output Frequency (GHz)	1.88	0.576–0.608	2.5–5.75	0.612–1.152
Reference Frequency (MHz)	80	32	125	36
Phase noise (dBc/Hz @1 MHz offset)	−122	−114	−115.9	−108
Jitter_rms_ (ps)(Integ. Range)	N/A	4.23(100 Hz~40 MHz)	0.34(10 kHz~40 MHz)	6.3(1 kHz~40 MHz)
Power consumption (mW)	55	10.5	5.3	0.95
Active area (mm^2^)	0.31	0.158	0.25	0.0648
FoM (dB)	N/A	−217	−242.4	−194.2
